# Contrast-enhanced ultrasound-guided sentinel lymph node biopsy in early-stage breast cancer: a prospective cohort study

**DOI:** 10.1186/s12957-023-03024-7

**Published:** 2023-05-08

**Authors:** Qiuxia Cui, Li Dai, Jialu Li, Yang Shen, Huijiang Tao, Xiaofeng Zhou, Jialei Xue

**Affiliations:** 1 Department of Thyroid and Breast Surgery, Changshu Hospital Affiliated to Nanjing University of Chinese Medicine, No. 6, Huanghe Road, Changshu, 215500 Jiangsu China; 2 Department of Ultrasonic Medicine, Changshu Hospital Affiliated to Nanjing University of Chinese Medicine, Changshu, China

**Keywords:** Sentinel lymph nodes (SLN), Sentinel lymph node biopsy (SLNB), Contrast-enhanced ultrasound (CEUS), SonoVue, Microbubbles, Breast cancer

## Abstract

**Objective:**

This study evaluated the identification efficiency of contrast-enhanced ultrasound (CEUS) for sentinel lymph nodes (SLN) to accurately represent the axillary node status in early-stage breast cancer.

**Method:**

In total, 109 consecutive consenting patients with clinically node-negative and T1-2 breast cancer were included in this study. All patients received CEUS to identify SLN before surgery, and a guidewire was deployed to locate SLN in those who were successfully explored by CEUS. The patients underwent sentinel lymph node biopsy (SLNB), and the blue dye was used to trace SLN during the surgery. The decision to perform axillary lymph node dissection (ALND) depended on the intraoperative pathological identification of SLN by CEUS (CE-SLN). The concordance rate of pathological status between CE-SLN and dyed SLN was calculated.

**Result:**

The CEUS detection rate was 96.3%; CE-SLN failed in 4 patients. Among the remaining 105 successful identifications, 18 were CE-SLN positive by intraoperative frozen section, and one with CE-SLN micrometastasis was diagnosed by paraffin section. No additional lymph node metastases were found in CE-SLN-negative patients. The concordance rate of pathological status between CE-SLN and dyed SLN was 100%.

**Conclusion:**

CEUS can accurately represent the status of axillary lymph nodes in patients with clinically node-negative and small tumor burden breast cancer.

**Graphical Abstract:**

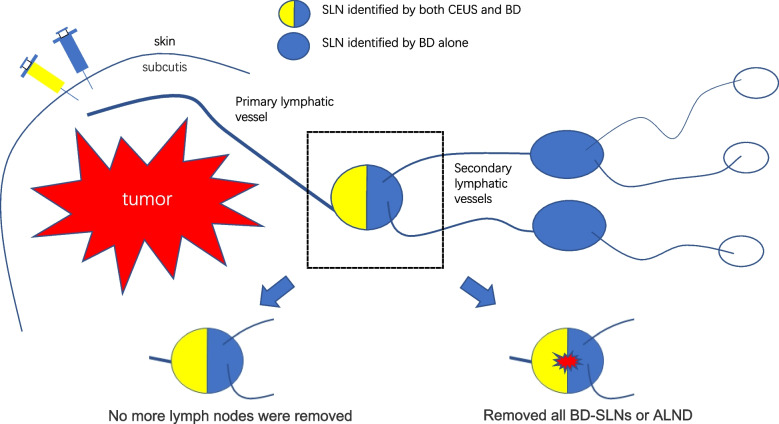

## Introduction

Sentinel lymph node biopsy (SLNB) is the preferred method for axillary node staging in clinically node-negative breast cancer patients. Blue dye and ^99^mTechnetium-sulfur colloid are the commonly used sentinel lymph node tracers. Studies have established that the dual-tracer method combining an isotope and dye has the highest tracing rate [1–3]. False negative rate (FNR) is an important index to evaluate the accuracy of SLNB. A B32 study recommended that for lower FNR, two or more SLN (sentinel lymph nodes) should be obtained in the operation, and the more the SLN obtained, the lower the FNR [1]. An ALMANAC study also reached a similar conclusion [2]. However, certain cases, where only one SLN was marked and obtained, make surgeons doubt the accuracy of the detection. Most such patients need to undergo extensive lymph node excision or axillary lymph node dissection for confident axillary staging, causing excessive surgery. In addition, sometimes, the tracer may recognize a lot of SLN. For instance, an ALMANAC study reported a maximum of 9 SLN [4]. Such a situation may occur due to tracer access reaching secondary lymphatic vessels, causing excessive axillary exploration and thereby leading to over-resection of lymph nodes. Although it reduces FNR, it increases the risk of postoperative complications, which is also contrary to the primary goal of SLNB.

Contrast-enhanced ultrasound (CEUS) is a novel lymph node tracing method, which has been used for SLNB surgery in several breast cancer studies [5–11]. The tracer used in CEUS is a microbubble contrast agent that enhances the ultrasonography of lymphatic vessels and lymph nodes. Given the higher molecular weight of the microbubble relative to the blue dye but with similar rapid entry into the lymphatic vessels, CEUS detects SLN within minutes after drug injection without flowing into the secondary lymphatic vessels immediately. With these features, SLN identified by CEUS are significantly less than those identified by the isotope-dye method [12–14]. Some studies have suggested that in cases of positive SLN, the SLN identified by CEUS must be included in positive nodes [9, 14]. In a previously published meta-analysis, we showed that the positive rate of sentinel lymph nodes identified by CEUS (CE-SLN) is more than six times higher than those by conventional methods [15]. The reason was that other secondary lymph nodes that were not identified by CEUS were eliminated as they were not real SLN and were the results of over-identification by conventional tracer methods. Importantly, in such node-negative patients, it could have been sufficient to remove the CEUS-identified SLN. Therefore, here, we conducted a prospective cohort study to investigate whether the pathological status of axillary lymph nodes can be accurately determined by CE-SLN identification alone.

## Methods

### Study design and population

This is a prospective, monocentric, single-arm, self-comparison, cohort study, and the clinical trial was registered at the maternal and child health research project of Jiangsu province, China (code: F201951). All patients underwent preoperative CEUS and intraoperative blue dye procedures. The concordance rate of pathological status between CE-SLN and dyed SLN was defined as the primary endpoint of the study. Patients’ inclusion criteria were as follows: (1) pathologically confirmed breast invasive carcinoma, (2) the tumor is unifocal with less than 5 cm in diameter, and (3) the clinical assessment is axillary lymph node-negative. The exclusion criteria were as follows: (1) diagnosis of distant metastasis before surgery, (2) history of surgery around the areola region or in the outer upper quadrant of the breast and axillary, including tumor excision biopsy, and (3) prior radiotherapy to the ipsilateral chest wall and axilla. The axillary node status was established by ultrasonography performed by two professional sonographers, and the subjects with suspicious lymph nodes were eliminated. The study was approved by the Institutional Ethics Committee, and all patients were asked for informed consent before surgery.

### CE-SLN

**Fig. 1:**
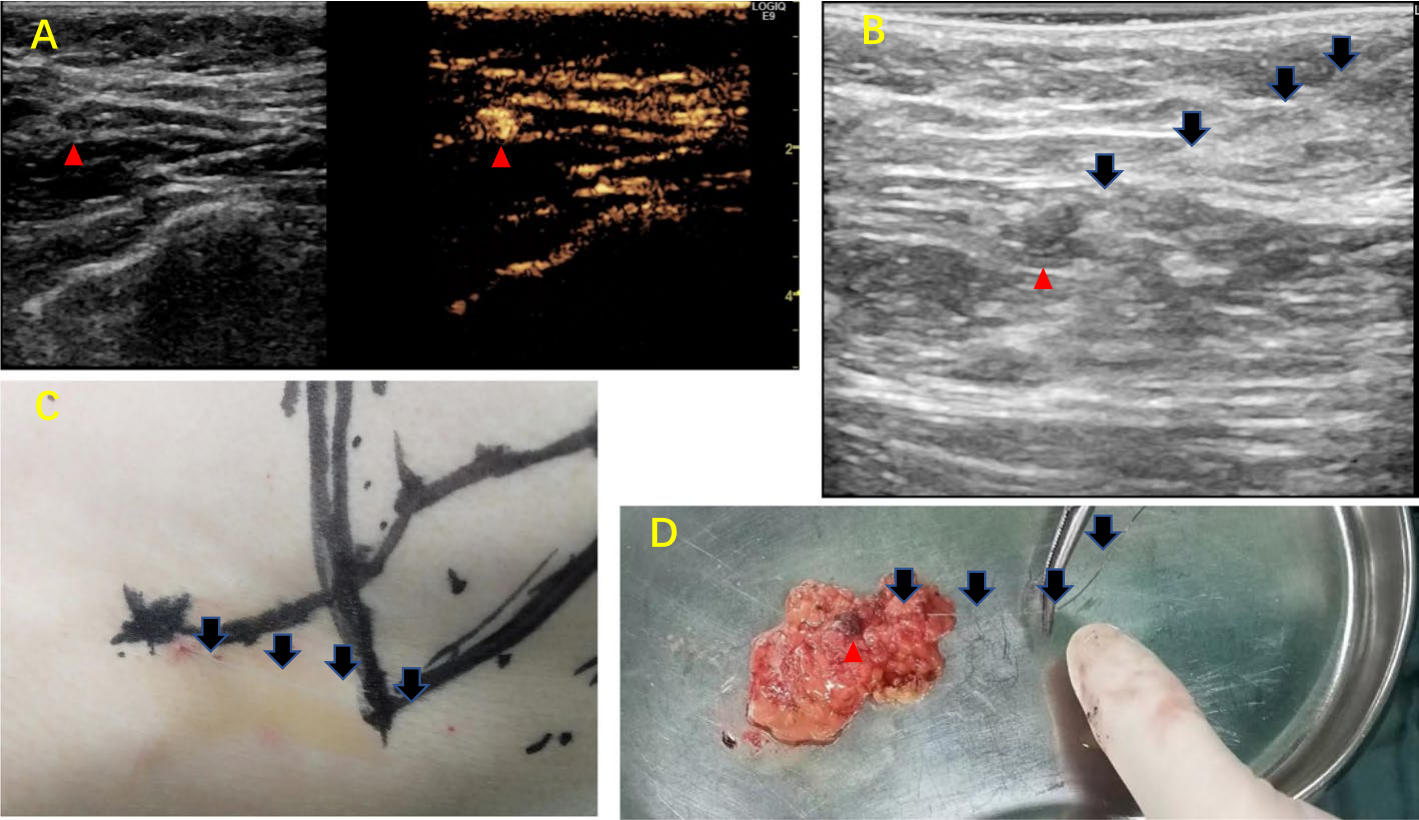
Operation process of identifying and locating the SLN by CEUS. **A** CEUS identify SLNS. **B**, **C** The hook wire locates the identified SLN. **D** Resected the positioned SLN along the hook wire (the red triangle indicates SLN; the black arrow indicates the hook wire)

CEUS was performed after general anesthesia of the patient and before surgery. The contrast agent was the microbubble reagent (SonoVue™ BRACCO Imaging, S.p.A, Milan, Italy), which was dissolved in 5 ml of saline (NaCl 0.9%). After repeated shaking, 0.5 ml liquid was respectively taken out and subcutaneously injected immediately at the areola edges at 12, 3, 6, and 9 o’clock and massaged for 1–3 min. The breast-to-axilla lymphatic flow was identified by CEUS using the Logiq E9 with the XDclear platform (GE Healthcare, Tokyo, Japan). The specific operation method is described in previous literature [16]. The operation was repeated if the enhanced lymph node was not explored within 5 min to confidently ensure the absence of SLN. After successfully exploring the enhanced SLN, the operation was repeated once more to confirm the number and location of SLN. Based on the direction of the lymphatic tube and the position of SLN, the positioning hook wire (20G, LW0077, BARD) was pierced into the lymph node under the guidance of ultrasound. Before beginning the study, a training set of 30 patients was operated on, which had a success rate of more than 90%, and was excluded from the study. The entire SLN identification and positioning process is shown in Fig. 1.

### Surgery

After the completion of SLN localization, the SLNB surgery was initiated immediately. Before the skin incision, 1 ml methylene blue was subcutaneously injected in the same location as the microbubble injection and kneaded gently for several minutes. Next, the surgeon made an incision of about 3 cm in length in the axilla according to the marked SLN, explored, and removed the SLN along the direction of the guide wire. The pathological status of CE-SLN was intraoperatively diagnosed by frozen section. While the intraoperative pathological result was on wait, the surgeon continued to explore and remove the remaining dyed lymph nodes. If the CE-SLN was pathological positive, axillary lymph nodes were dissected, and if the CE-SLN was pathological negative, except for the remaining dyed lymph nodes, peripheral non-dyed lymph nodes were also removed until the number of SLNs reached 6. These supplementary SLNs and axillary dissection specimens were directly diagnosed by paraffin sections and no intraoperative frozen sections. The operation time was recorded as the CE-SLN and the BD-SLN (blue dye-identified SLN) operation times, respectively. CE-SLN operation time included the period from skin incision to excision of CE-SLN. Also, the time of each dyed lymph node excision was recorded. BD-SLN operation time is the time taken from the skin incision to the removal of the last dyed lymph node.

### Statistical analysis

All data were recorded as an EXCEL database (Microsoft, Redmond, WA, USA). Continuous variables are reported as mean and median numbers (e.g., node numbers and operation times); categorical variables are reported as numbers and percentages. SPSS statistical software version 18.0 was used (SPSS Inc. Chicago, IL, USA) for statistical analysis. Continuous variables between procedures were compared with Mann–Whitney *U* test, while categorical variables were subjected to Fisher exact test. The concordance rate is represented as the percentage of concordant patients. Data with a *P*-value < 0.05 were considered statistically significant.

## Results

### Preoperative detection rate of CEUS-SLN

**Fig. 2:**
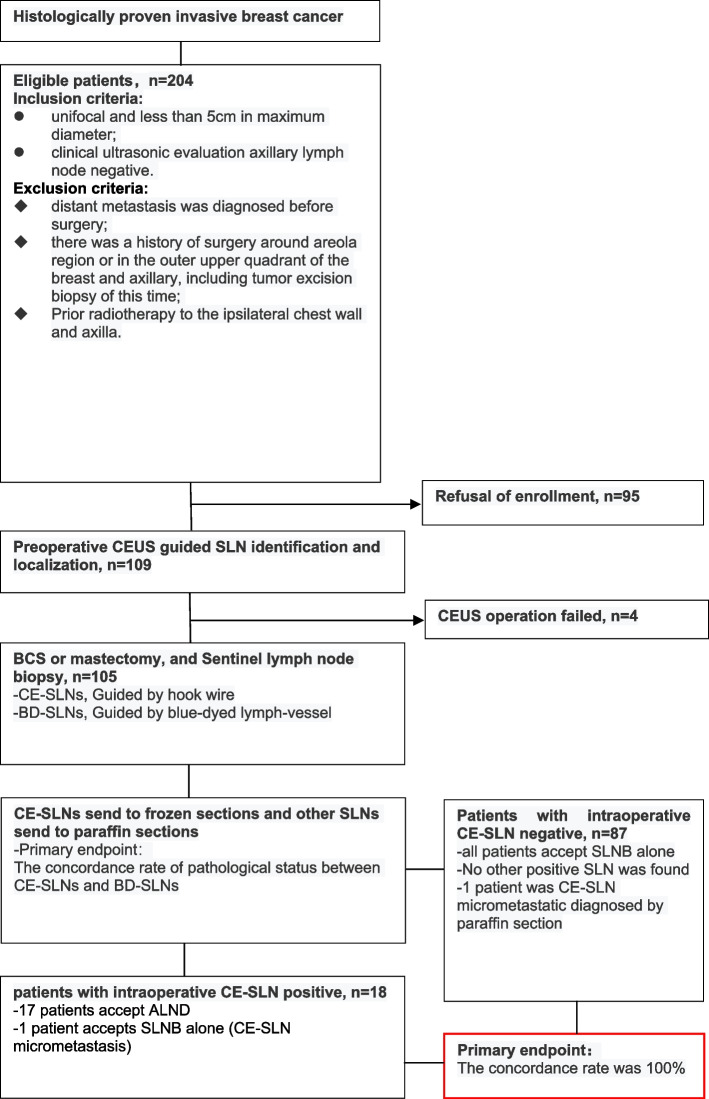
Study flowchart. BCS, breast-conserving surgery; BD, blue dye; CEUS, contrast-enhanced ultrasound; SLN, sentinel lymph node; CE-SLN, sentinel lymph nodes identified by CEUS; BD-SLN, sentinel lymph nodes identified by BD

From May 1, 2020, to May 31, 2022, in total, 204 female patients were found eligible, of which 109 agreed to the protocol and formed the initial study population. Out of the 109 patients, 105 were identified by CEUS-SLN, while the other 4 only accepted the dye method. Therefore, the detection rate of CEUS was 96.3%. Among the 105 successful patients, 72 patients were located with one CE-SLN, and 33 patients were located with two CE-SLN: the mean number of CE-SLN was 1.3. The clinical characteristics of patients are listed in Table 1, and the complete study process is shown in Fig. 2.

**Table 1:** Clinical characteristics of the initial population

Characteristics	Number of patients (%)
**Age**	
< 50 years	34 (31.2%)
≥ 50 years	75 (68.8%)
**T stage**	
T1	91 (83.5%)
T2	18 (16.5%)
**Grade**	
I	2 (1.8%)
II	68 (62.4%)
III	39 (35.8%)
**Histology**	
Ductal carcinoma	99 (90.8%)
Lobular carcinoma	3 (2.8%)
Papillary carcinoma	5 (4.6%)
Mucinous adenocarcinoma	2 (1.8%)
**ER/PR status**	
ER positive and/or PR positive	85 (78.0%)
ER and PR negative	24 (22.0%)
**Her2 status**	
Positive	20 (18.3%)
Negative	89 (81.7%)
**Subtype**	
Luminal A/B (Her2-negative)	78 (71.6%)
Her2-enriched	20 (18.3%)
Triple-negative	11 (10.1%)
**Pathological N status**	
Negative	87 (79.8%)
Positive	22 (20.2%)
≥ 2 positive nodes	5 (4.6%)
**Surgical management**	
Mastectomy	86 (78.9%)
Breast-conserving surgery	23 (21.1%)
**Method of diagnosis**	
Core needle biopsy	74 (67.9%)
Excision biopsy^a^	35 (32.1%)

^a^Excision biopsy was permitted when the tumor is located outside the outer upper quadrant and areola region

### Primary endpoint

Based on the records of SLNB surgery, BD-SLN were successfully found in the subsequent SLNB surgery in all 105 patients with successful CEUS operations: the mean number of BD-SLN was 2.5, including all of the CE-SLN. The pathological node metastasis status of the 105 patients showed that 86 were axillary node-negative, and 19 were positive. Among the 19 node-positive patients, at least one CE-SLN was metastatic for every patient, and 17 of the node-positive patients were CE-SLN positive alone with a concordance rate of 100%. Notably, two of the patients had CE-SLN micrometastases (one of them was found in paraffin pathology), and neither of them received ALND. All 19 node-positive patients are listed in Table 2. Among the 4 CEUS unsuccessful patients, 3 failed even in the dye method, while the final pathological results showed that they had lymph node metastasis.

**Table 2:** Details of node positive patients

CASE No	Age (years)	Grade	Surgical management	Maximal diameter (mm)	Number of CE-SLN	CEUS operation times	Number of dyed SLN	Intraoperative pathology of CE-SLN (positive number/total number)	Size of CE-SLN metastasis (macro/micro)	Pathological status of other ALNs or SLNs
Case4^a^	85	II	Mastectomy	20	1	1	3	0/1	Micro	0/5
Case10	64	II	Mastectomy	15	2	2	2	1/2	Macro	0/16
Case19	42	III	Mastectomy	20	2	2	2	1/2	Macro	0/16
Case29	48	III	BCS	25	1	2	2	1/1	Macro	1/18
Case36	51	II	BCS	15	1	1	3	1/1	Macro	0/7
Case39	64	II	Mastectomy	20	1	1	1	1/1	Macro	0/17
Case42	59	III	Mastectomy	20	2	1	2	1/2	Macro	0/13
Case45	48	II	BCS	30	1	1	1	1/1	Macro	0/12
Case59	73	II	Mastectomy	19	1	1	3	1/1	Micro	0/5
Case64	63	II	Mastectomy	17	2	2	3	1/2	Macro	0/19
Case72	46	II	Mastectomy	15	1	2	2	1/1	Macro	0/15
Case73	42	III	Mastectomy	20	1	2	2	1/1	Macro	0/13
Case76^b^	27	III	BCS	20	1	2	3	1/1	Macro	2/17
Case80	50	II	BCS	15	1	1	2	1/1	Macro	0/13
Case88	48	II	BCS	22	1	1	1	1/1	Macro	0/15
Case92	63	II	Mastectomy	20	1	1	2	1/1	Macro	0/20
Case97	57	III	Mastectomy	23	2	1	2	1/2	Macro	0/12
Case104	60	II	Mastectomy	25	1	2	1	1/1	Macro	0/18
Case105	45	III	Mastectomy	18	1	2	2	1/1	Macro	0/19

*BCS* Breast-conserving surgery

^a^The intraoperative frozen section was negative, and CE-SLN micrometastasis was found in the paraffin pathology

^b^In this patient, the two positive non-CE-SLNs were dyed, and one of them was micrometastasis

### Analysis of CEUS operation records

CEUS operation records showed that 84 patients were successfully displayed and positioned SLN with only one injection of contrast agent; only 11.9% (10/84) patients were node positive. On the contrary, 21 patients required the second injection for SLN positioning, and 42.8% (9/21) were node positive. The latter group had a significantly higher risk of lymph node metastasis than the former. Among node-positive patients, 42.1% (8/19) had only CE-SLN dyeing, compared with 20.9% (18/86) of node-negative patients. The results from the above analysis are listed in Table 3. The median number of dyed lymph nodes was 2 in node-positive patients and 3 in node-negative patients. The mean operation time of CE-SLN was 415 ± 134 se, which was less than that of BD-SLN (536 ± 201 s).

**Table 3:** Comparative analysis of operation record data

	Node positive	Node negative	OR (95%CI)	*P* value
CEUS operation times				
1	10	74	0.278 (0.130–0.596)	.003
2	9	12		
Dyed node except CE-SLN				
Yes	11	68	0.453 (0.204–1.003)	0.101
No	8	18		

## Discussion

Since the 1990s, the use of dye and isotope has been the widely accepted tracer method in SLNB staging of breast cancer [17–19]. Although the isotopic method is considered the most accurate, it is limited by its high cost and potential radiological hazards. Likewise, even though the dyeing method is simple and low-cost, it causes tattoo effects and allergic reactions [20, 21]. In patients with nipple-sparing mastectomy and immediate implant-based reconstruction, the dye-induced periareolar coloration increases the risk of nipple necrosis [22]. CEUS-guided SLNB has been reported for more than a decade, especially, correlative research has increased year by year in the last five years. Using an ultrasonic enhanced agent, with the assistance of an ultrasound professional doctor, a clinical surgeon can accurately know the location of SLN before the surgery: the method is as simple as the dyeing method. SLNB becomes further easier with wire positioning, reducing any possible damage.

In several prospective studies, the detection rate of CEUS-localized SLN has been more than 90%. For instance, Li et al. reported an SLN detection rate of 98.23%, which included 453 patients [10]. Zhong et al.'s study included 126 patients and had an SLN detection rate of 100% [23]. In this study, the SLN detection rate was 96.3%, which is consistent with previous studies and not inferior to the isotope and dye method. In addition, consistent with previous reports [12–14], the average number of CEUS identified SLN in our study was 1.3, which is significantly smaller than that obtained by the dye method. This was mainly due to differences in the molecular weight of the two tracers [6, 7]. A smaller tracer with a longer tracer duration is more likely to enter the secondary lymphatic vessels, increasing the removal of more non-SLN and postoperative complications. Previous studies, like in B32 and ALMANAC, also suggested that although SLNB significantly reduces the risk of upper limb complication compared with ALND, about 8–41% of patients experience upper limb paresthesia and other complications [24]. Retrospective studies and meta-analyses have demonstrated that there is always some morbidity in the upper limb after SLNB surgery; the most common is axillary pain, which torments more than 10% of patients [25–27]. The main cause may be the excessive exploration of SLN. A previous study suggested that, in a small minority of patients, SLN for the breast and upper limbs may be at the same station [28]. The CEUS method can find the real SLN more quickly and accurately, reducing the exploration of other secondary lymph nodes and, in turn, lowering upper limb complications. Currently, several RCT studies about omitting sentinel lymph node biopsy are underway [29]; however, the persuasive results and conclusions need a long follow-up, which practically is not possible in current clinical practice. Therefore, like the CEUS method, reducing the surgical trauma of SLNB while ensuring diagnostic accuracy is a more practical approach at this stage.

In this study, all preoperatively located CE-SLN were dyed. However, some previous reports showed that some CEUS-identified SLN cannot be dyed [9, 30], which may be related to the lymphatic drainage pattern in such patients. The lymphatic drainage pattern from the mammary gland to the axilla can be divided into four modes: (1) a single primary lymphatic vessel corresponds to a single SLN, (2) a single primary lymphatic vessel after branching corresponds to two or more SLN, (3) multiple primary lymphatic vessels correspond to multiple SLN, and (4) multiple primary lymphatic vessels when aggregated correspond to a single SLN [13]. (1) and (2) account for more than 80% of the total, and the third is the rarest. So, if CE-SLN remains undyed, the first lymph node corresponding to the dyed primary lymphatic vessel must be removed to minimize the FNR.

In our study, three quarters of the patients who failed in CE-SLN identification were node metastatic. Further analysis of CEUS operating records found that, in all lymph node-positive patients, 47.4% belonged to the secondary operation group with an SLN-positive rate of 42.8%, which may be related to the poor flow of lymphatic tubes in these patients [31]. Given this data, in clinical practice, the success rate of SLNB can be well predicted by the CEUS method, while in patients with CEUS failure, double tracing may be necessary to increase the success rate. On the contrary, in patients with successful SLN localization after a single CEUS operation, most of them were node-negative or had only a mild tumor burden in the SLN. In all, CEUS can accurately predict the axillary state or lymph node load before surgery.

Compared with the single dye method, the operation time of CEUS-guided SLNB is significantly reduced. Furthermore, the operation time of the dye method in this study was shortened because of the guidance from the guide wire, suggesting that the real operation time of the single dye method can be longer. CEUS can also shorten the learning curve of surgeons and improve the accuracy of SLN exploration [32]. In this study, the false negative rate of CE-SLN was 0%. Although these patients did not undergo ALND, at least six lymph nodes including all dyed nodes were removed. Notably, previous studies have shown that the risk of FNR is very small when more than 5 lymph nodes are removed [1, 2].

Nonetheless, this study had some limitations. Firstly, 83.5% of the patients were in early breast cancer stage T1; the application value of the CE-SLN in patients with larger tumor diameters needs to be further studied. This study only investigated the methodological feasibility and the effect on long-term recurrence was lacking in data. Meanwhile, the impact of CE-SLN on upper limb function needs to be further evaluated to validate its superiority over traditional methods. The intraoperative tracer in this study was the blue dye, which is not the best single tracer and has the tattoo effect and other defects. CEUS combined with isotope may overcome the defects of the dye method, which can also be used to verify the results of this study. Finally, more clinical studies would help confirm these results.

## Conclusion

In clinically node-negative breast cancer patients, SLNB under CEUS guidance is a high-efficiency new method, avoiding probable excessive exploration of SLNs. Nonetheless, more clinical studies are needed to confirm these results.

## Data Availability

All data analyzed in this study are included in this published article.
